# A Treatise on Food and Dietetics

**Published:** 1874-08

**Authors:** 


*A Treatise on Food and Dietetics, Physiologically and Therapeutically Considered.* By F. W. Pavy, M. D., F. R. S. Philadelphia: Henry C. Lea, 1874. Buffalo: T. Butler & Son.

Dr. Pavy is already somewhat well known as an author of a work upon the Disorders of Digestion and their Treatment. The present volume is somewhat imposing in size when the subject under discussion is taken into view, it containing nearly six hundred pages. The work undertaken by the author is very well carried out, and much valuable information can be gleaned from his treatise; there are points, however, which could have been omitted and others considered in their places which would have added much to the value of the book. A little additional light upon the relative value of certain foods, as broths, etc., for invalids would have proved very acceptable to medical readers. On the whole, however, the work is one of much value, and we are pleased to learn that the author has taken a little more pains to inform himself upon the nature of American animal and vegetable foods than did Dr. Letheby, the author of a somewhat similar English work, upon a smaller scale.

*Archives of Ophthalmology and Otology.* By Prof. H. KNAPP, M. D., of New York, and Prof. S. Moos, M. D., of Heidelberg. Quarterly. New York: Wm. Wood & Co. Vol. I, No 1.

This valuable Journal has been changed from a semi-annual to a quarterly publication, and in order to facilitate the prompt appearance of the American Edition, Drs. E. Gruening, of New York, and Clarence J. Blake, of Boston, have been added to the Editorial Staff. The present number contains several valuable contributions to Ophthalmology and Otology, some of the articles being illustrated by fine lithographic plates. The reviews of Otology by Dr. Blake, and of Ophthalmology by Drs. Gruening and Knapp, will be found to be comprehensive and succinct statements of the recent advances in these departments. The publishers inform us that the subscription priee has been reduced from seven to five dollars per year.

